# Engineering the plasmon modes of a confined electron gas

**DOI:** 10.1515/nanoph-2023-0795

**Published:** 2024-02-15

**Authors:** Andrew Haky, Angela Vasanelli, Konstantinos Pantzas, Yanko Todorov, Grégoire Beaudoin, Gilles Patriarche, Isabelle Sagnes, Carlo Sirtori

**Affiliations:** Laboratoire de Physique de l’Ecole normale supérieure, ENS, Université PSL, CNRS, Sorbonne Université, Université Paris Cité, 75005 Paris, France; Université Paris-Saclay, CNRS, Centre de Nanosciences et de Nanotechnologies, 91120 Palaiseau, France

**Keywords:** quantum plasmonics, semiconductor plasmonics, plasmon hybridization, Berreman mode, bulk plasmons, quantum engineering

## Abstract

The volume plasmon modes of a confined electron gas are engineered in a step-like semiconductor potential, which induces the formation of adjacent regions of different charge density. Each region supports spatially localized collective modes. Adjacent modes are theoretically demonstrated to couple, forming delocalized modes, which are well-described with a hybridization picture. Exploiting the thin-film Berreman effect, the engineered plasmon modes are directly observed in optical measurements. Using a quantum microscopic theory, the asymmetry of the single-particle electronic states is shown to be directly imprinted on the nonuniform polarization of the collective modes.

## 1 Introduction

Plasmons are excitations of collective modes of the electron gas, which correspond to self-sustaining oscillations of charge density [1]. Their strong interaction with light and their appealing ability to concentrate far-field energy into subwavelength volumes has long motivated their study.

More recently, an intense interest has arisen in investigating plasmons specifically in the *quantum regime* [2], [3], [4], in which the electronic potential cannot be neglected and the spatial dependency of the polarization of the electron gas must be considered. In this regime, a nonlocal, or wavevector-dependent treatment, is required [5], [6], [7]. These studies have mostly been focused on the investigation of metallic nanoparticles and dimers [4], where size confinement and tunneling can play an important role. Different theoretical approaches have been employed in order to account for these quantum effects in the description of plasmons. Electronic structure methods [8], including density-functional theory and many-body perturbation theory, calculate the electronic structure of the constituent atoms of the nanoparticle, the quantization of their energy levels, and the screening effects. These methods can thus accurately predict electronic many-body excitations but are computationally very demanding for nanoparticles larger than a few nanometers. Alternatively, hydrodynamic models are able to describe structures of several hundreds of nanometers in size, including some quantum effects via an electron pressure term. More recently, quantum hydrodynamic theory for the spatial dependence of the electron density and for nonlocality effects has been developed [9].

Our investigation proposes the use of electronic states obtained within the envelope function approximation as the basis to describe plasmons in a semiconductor nanostructure. Indeed, the plasmonic excitations are constructed from the single electron wavefunctions, by considering the dipole–dipole coupling between all possible electronic transitions of the structure [10], [11]. This approach allows collective effects, quantum confinement, and tunneling to be treated on the same grounds, and directly includes nonlocality effects [12].

It has long been an intriguing proposition to engineer plasmon modes. To date, efforts have been limited to studying metallic systems in the *classical regime*. Two concepts emerged from these studies: first, novel bright modes can be observed when the system symmetry is broken [13], and second, that the plasmon modes of complex structures can be described as resulting from the hybridization of simpler elementary modes [14], [15], [16].

Here, we experimentally demonstrate a robust engineering of plasmon modes in the *quantum regime* by adopting a radically different approach based on volume plasmons in highly doped subwavelength semiconductor heterostructures [17], [18], [19]. We exploit the precisely controllable, one-dimensional confining potential of the semiconductor heterostructure as a degree of freedom, similarly to what is routinely done to engineer the band structures of quantum optoelectronic devices.

We demonstrate that by modifying the symmetry of the confining potential, the oscillator strength can be redistributed among the collective modes, thus transforming dark modes into bright modes, and that the spatially nonuniform polarization of the modes can be manipulated. We show that the two concepts that emerged from plasmon engineering in the classical regime cited above hold true in the quantum regime. Moreover, we establish that they are consequences of the fact that the (many-body) collective modes inherit the symmetry of the wavefunctions of their constituent (single-particle) electrons.

## 2 The step potential

**Figure 1:**
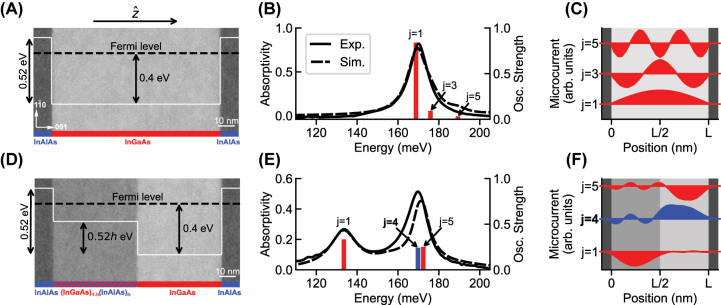
Plasmon modes in square well and step well potentials. The square well and step well potentials are overlaid on TEM images of the corresponding structures in (A) and (D). The height of the step is linearly proportional to the InAlAs content *h* in the quaternary alloy region. Absorptivity spectra and calculated oscillator strengths of the plasmon modes are plotted for the square well in (B) and for the step well with *h* = 0.3 in (E). The microcurrents associated with the modes *j* denoted in (B) and (E) are plotted in (C) and (F), respectively. In (F), the darker shaded region corresponds to the quaternary, or step side, of the structure.

Our approach is summarized in Figure 1 in which the plasmon modes observed in a square well potential (upper panels) are compared with those observed in a purposefully engineered step potential (lower panels). Confined in a square well, such as the 100 nm wide heavily doped (volume electronic density of *N*_v_ = 2 × 10^19^ cm^−3^) In_0.53_Ga_0.47_As/Al_0.48_In_0.52_As quantum well shown in the TEM image of Figure 1(A), the electron gas supports a quantized dispersion of longitudinal modes in the direction of confinement, $\hat{z}$ , which can be excited by *p*-polarized, obliquely incident light [12], [20], [21], [22], and which can be conveniently indexed with odd-*j*, for reasons which will become clear below.

In Figure 1(B), experimental and simulated absorptivity spectra of the square well structure are plotted for *p*-polarized light incident at 18° inside the semiconductor substrate (*n* = 3.1). The experimental spectrum was found as one minus the measured reflectivity, after a gold mirror was deposited on the sample surface to ensure zero transmission [additional experimental details are provided in Section 1 of the Supplementary Material]. The simulated spectrum was calculated by solving the quantum Langevin equations for the plasmon modes in the input–output formalism [23], using a phenomenological value for the nonradiative linewidth. In the spectra, a single dominant peak is observed, identified as the Berreman *j* = 1 mode [18], [24], [25], which corresponds to the excitation of a macroscopic dipole in the $\hat{z}$ direction.

The plasmon modes of an electron gas confined in an arbitrary potential can be calculated as the eigenmodes of a Hamiltonian describing the single-particle excitations of the electron gas and their dipole–dipole Coulomb interaction [10], [11]. The *z* dependence of the polarization associated with mode *j* is captured in the current density distribution *J*_*j*_(*z*), which we call a *microcurrent* [additional details on the calculation of the plasmon modes and their properties are found in Section 2 of the Supplementary Material]. The oscillator strength $F_{j}$ of mode *j* is found to be directly linked to *J*_*j*_(*z*):

(1) $$F_{j}\propto {\left|\overset{+\infty }{\underset{-\infty }{\int }}J_{j}\left(z\right)\;dz\right|}^{2}$$

In Figure 1(B), the oscillator strengths of the plasmon modes in the square well, normalized to a total of one, are plotted with bars at the mode energy, and in Figure 1(C), the normalized microcurrents associated with each odd-*j* mode are plotted. It is evident from the form of the microcurrents that each mode *j* corresponds to a standing polarization wave $J_{j}\left(z\right)\propto \sin\left(k_{j}z\right)$ with longitudinal wavevector *k*_*j*_ = *jπ*/*L*, where *L* is width of the well. As observed in the experiment, the *j* = 1 mode captures the vast majority of the oscillator strength. As a consequence of Eq. (1), the oscillator strength of the other *j* > 1 modes declines as *j* ^−2^ with respect to the *j* = 1 mode, and there can be no bright even-*j* modes, as the integral would be zero. As a consequence, only the *j* = 1 mode is observed in the experimental spectrum of Figure 1(B).

The above rules can be relaxed by modifying the symmetry of the confining potential. We study the step well potential plotted in Figure 1(D), realized by growing 50 nm ternary InGaAs and 50 nm quaternary (InGaAs)_1−*h*_(InAlAs)_*h*_ layers between two InAlAs barriers. By varying the InAlAs content *h* in the quaternary layer, the height of the step can be continuously varied [26]. The entire 100 nm region between the InAlAs barriers is heavily doped with a volume electronic density of *N*_v_ = 2 × 10^19^ cm^−3^.

In Figure 1(E), experimental and simulated absorptivity spectra of an *h* = 0.3 step well structure are plotted for an 18° angle of incidence, along with the oscillator strengths of the calculated modes. The absorptivity spectra are dramatically modified from the square well case: two peaks of comparable amplitude are observed. Remarkably, as compared to the square well case, a significant fraction of the oscillator strength is redistributed to the usually dark *j* = 4 mode and to the *j* = 5 mode, such that these modes acquire oscillator strengths comparable to that of the *j* = 1 mode. Being separated within the linewidth, both the *j* = 4 and *j* = 5 modes contribute to the single peak observed at 170 meV.

The redistribution of the oscillator strength results from the modified forms of the microcurrents *J*_*j*_(*z*), plotted in Figure 1(F), which enter the integral of Eq. (1): the positive and negative lobes of the even *j* = 4 microcurrent no longer strictly cancel, and the odd *j* = 5 microcurrent is no longer strictly mirror symmetric with respect to the well center. While not plotted, there are also *j* = 2 and *j* = 3 modes that have finite but negligibly small oscillator strength. As such, these modes are not observed in the experimental spectra.

**Figure 2:**
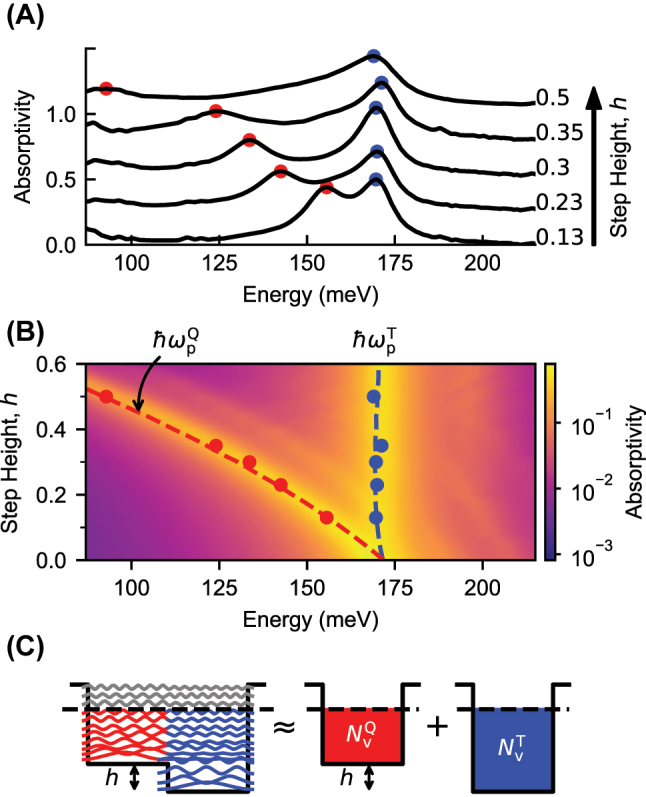
Absorption in a step potential. (A) Experimental absorptivity spectra of step potential structures are plotted, linearly offset by the step height *h*. (B) Calculated absorptivity spectra for step potentials of varying *h* are plotted using a logarithmic color scale. The experimental resonances from (A) are plotted with scatter points. Effective plasma frequencies, calculated as described in the text, are plotted with dashed lines. (C) The square moduli of the wavefunctions of a 50 nm wide step potential are plotted at their respective energies. The form of the wavefuctions imply that the step structure can be mapped to two separate square wells.

We experimentally studied step potential structures in which the step height was varied. The absorptivity spectra of the structures, measured at an 18° angle of incidence inside the gold plated semiconductor, are plotted in Figure 2(A) with an offset proportional to the step height, *h*. For all step heights, two peaks are observed. As the step height is varied, the higher energy peak remains fixed near 169 meV, whereas the lower energy peak redshifts with increasing step height.

In Figure 2(B), the experimentally observed peaks (scatter points) are plotted over simulated absorptivity spectra (logarithmic color scale) calculated for varying step height, but for a fixed Fermi level of 400 meV. The behavior of the experimentally observed peaks is well-reproduced by the simulated spectra.

It can be easily understood that, for a fixed Fermi level, as the step height is raised, the electronic density over the step side of the structure is reduced since the density of states below the Fermi level decreases, while the electronic density on the ternary side of the structure remains nearly constant, since the density of states is little changed.

Consequently, the behavior of the higher and lower energy peaks can be well described by introducing effective plasma frequencies $\omega_{\text{p}}^{\text{T}}=\alpha \sqrt{N_{\text{v}}^{\text{T}}}$ and $\omega_{\text{p}}^{\text{Q}}=\alpha \sqrt{N_{\text{v}}^{\text{Q}}}$ , where $\alpha =\sqrt{e^{2}/\left(m^{*}\varepsilon_{\infty }\epsilon_{0}\right)}$ with m* = 0.095*m*_0_ and *ɛ*_∞_ = 3.1, and for which $N_{\text{v}}^{\text{T}}$ and $N_{\text{v}}^{\text{Q}}$ are volume electronic densities evaluated over the respective ternary or quaternary regions of the step structure. Over the quaternary region [0, *L*/2], the volume electronic density is found as:

$$N_{\text{v}}^{\text{Q}}=\frac{1}{L/2}\overset{\frac{L}{2}}{\underset{0}{\int }}\left(\underset{i}{\sum }n_{i}{\left|\psi_{i}\left(z\right)\right|}^{2}\right)\;dz$$

where *n*_*i*_ and *ψ*_*i*_(*z*) denote the sheet density and wavefunction of subband *i* [additional details can be found in Section 3 of the Supplementary Material]. The density $N_{\text{v}}^{\text{T}}$ is found similarly by evaluating the integral over the region [*L*/2, *L*].

The effective plasma frequencies $\omega_{\text{p}}^{\text{T}}$ and $\omega_{\text{p}}^{\text{Q}}$ are plotted with dashed lines in Figure 2(B) and are found to be in remarkable agreement with the measured peaks. In the limit as the step height approaches the Fermi level, the volume electronic density on the step side of the structure goes to zero, and consequently, $\omega_{\text{p}}^{\text{Q}}\to 0$ .

Why does the effective plasma frequency description work so well to describe the experimentally observed peaks? Returning to Figure 1(E) and (F), it is observed that the microcurrent of the *j* = 1 mode, responsible for the oscillator strength of the lower energy peak, is almost entirely localized to the quaternary region of the structure. Likewise, the microcurrents of the *j* = 4 and *j* = 5 modes, together responsible for the oscillator strength of the higher energy peak, are largely localized to the ternary side of the structure, over which they have a single lobe and, thus, take on the form of a *j* = 1 mode. The density of charge that participates in each mode is, thus, naturally related to the density of charge of the spatial region over which the excitation extends.

## 3 Hybridized modes

It is remarkable that the lower energy *j* = 1 (or $\omega_{\text{p}}^{\text{Q}}$ ) mode is well confined to the quaternary region of the structure, despite the fact that the constituent electrons participating in the excitation have energies greater than the step height, as well as wavefunctions completely delocalized across the whole structure. The localization of both the lower and higher energy modes is a consequence of the fact that, over either side of the structure, the wavefunctions have nearly the same symmetry as expected for a square well. This is evident in Figure 2(C), where the square moduli of the wavefunctions for a step structure of 50 nm total width (chosen for legibility) are plotted. Consequently, each side of the step structure can be mapped to a separate square well, provided the difference between the Fermi level and the conduction band minimum of the respective step region is maintained. The same series of *j* modes that arise in a square well are therefore supported on either side of the step structure. We now proceed to show that *j* modes localized to opposite sides of the step structure can couple, when resonant, to form hybridized modes which extend across the whole structure.

Let *j*_T_ and *j*_Q_ be quantum numbers for the quantized dispersion of plasmon modes excitable in separate 50 nm ternary and quaternary wells, or on respective sides of the 100 nm step potential. The quantized dispersion of plasmon modes *j* in a square well is described by the following expression [12]:

(2) $$\Omega \left(j\right)=\sqrt{\omega_{\text{p}}^{2}+{\left(j\omega_{0}\right)}^{2}},$$

in which *ω*_0_ is the average level spacing between the confined single-particle electronic states. For 50 nm ternary and quaternary wells, $\omega_{0}\ll \omega_{\text{p}}^{\text{T}}\approx \omega_{\text{p}}^{\text{Q}}$ , and thus as already established, the *j*_Q_ = 1 and *j*_T_ = 1 modes correspond to the $\omega_{\text{p}}^{\text{Q}}$ and $\omega_{\text{p}}^{\text{T}}$ modes, provided the *j*_T_ and *j*_Q_ modes are fully decoupled.

**Figure 3:**
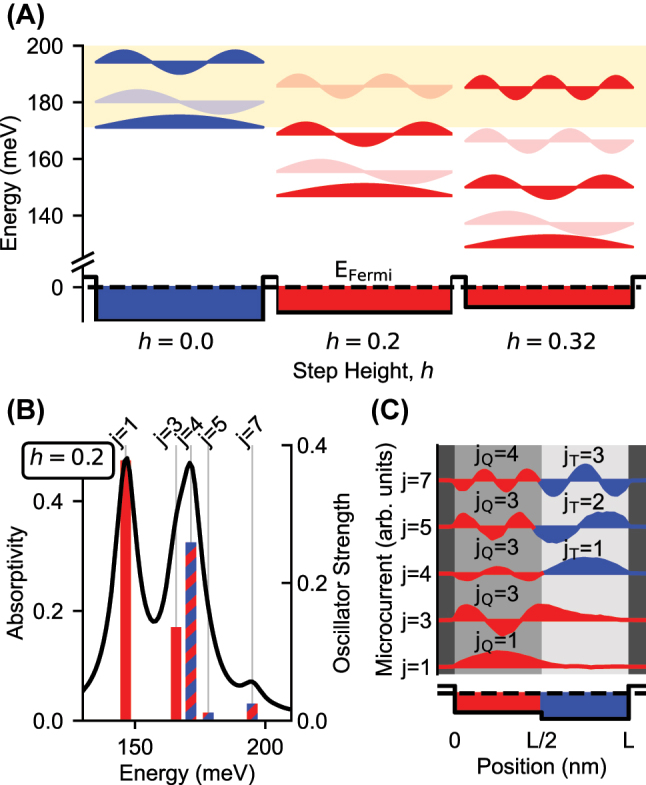
Hybridization of plasmon modes. (A) The plasmon modes of 50 nm square wells, which can be mapped to the ternary (blue) or quaternary (red) sides of the step structure, are plotted for various values of the step height. The microcurrents are plotted at their respective mode energies. Dark, even-*j* modes are plotted with lighter shade. The yellow shaded region indicates the energy range over which modes hybridize. (B) The absorptivity spectrum and oscillator strengths for a step structure with step height *h* = 0.2 are plotted. Hybridized modes are indicated with bars of alternating color. (C) Microcurrents associated with each mode *j* labeled in (B) are plotted.

In Figure 3(A), the spatial distribution of plasmon modes is sketched for three 50 nm quantum wells, which can be mapped to the ternary (blue) or quaternary (red) sides of the step structure. As the step height is increased from *h* = 0.2 to *h* = 0.32, the dispersion of the *j*_Q_ modes uniformly shifts to lower energy. This is described by Eq. (2), since as *h* is increased, *ω*_0_ varies little, while $\omega_{\text{p}}^{\text{Q}}$ uniformly decreases.

Above the lowest energy *j*_T_ = 1 mode of the ternary side (above $\hbar \omega_{\text{p}}^{\text{T}}\approx$ 170 meV), the *j*_T_ and *j*_Q_ modes can be in resonance. Over this energy range, shaded in light gold in Figure 3(A), the plasmon modes of the step potential result from a coupling, or *hybridization*, of *j*_T_ and *j*_Q_ modes. At energies below this range, the plasmon modes of the step potential are exclusively *j*_Q_ modes.

In Figure 3(B), the calculated absorptivity spectrum for a step structure with step height *h* = 0.2 is plotted. On the same axis, the oscillator strengths of the plasmon modes from the full step structure are plotted with bars on a linear scale. The microcurrents associated with the modes are plotted in Figure 3(C). The modes (bars) and their associated microcurrents are labeled by the integer *j*, defined as the total number of lobes observed in the microcurrent across the entire step structure.

The *j* = 1 and *j* = 3 modes, lying below $\hbar \omega_{\text{p}}^{\text{T}}$ and plotted with solid red bars, have microcurrents corresponding to *j*_Q_ = 1 and *j*_Q_ = 3 modes, localized to the quaternary side of the step structure.

The *j* = 4, *j* = 5, and *j* = 7 modes, plotted with multicolored bars, are hybridized modes for which *j* = *j*_Q_ + *j*_T_. The microcurrents associated with these modes have the form of *j*_Q_ modes over the quaternary region and *j*_T_ modes over the ternary region. Of note, the *j* = 5 and *j* = 7 modes are shown in Figure 3(C) to result from the hybridization of a dark mode with a bright mode (an even and odd couple of *j*_Q_ and *j*_T_).

**Figure 4:**
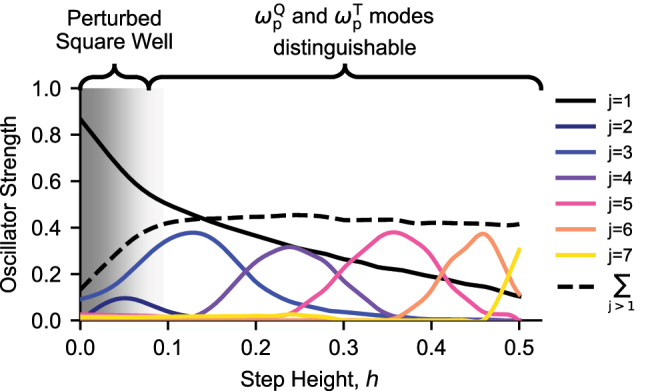
Distribution of oscillator strength as a function of step height. The oscillator strength of each mode *j* of the 100 nm step well potential, normalized so that the total oscillator strength is one, is plotted as a function of the step height *h*. The sum of the curves from *j* = 2 to *j* = 7 is plotted with a dashed line.

As shown in Figure 3(A), as *h* is increased, the *j*_Q_ mode that resonantly couples to the *j*_T_ = 1 mode successively changes from *j*_Q_ → *j*_Q_ + 1 → *j*_Q_ + 2 … . Since the *j*_T_ = 1 mode is fixed to $\hbar \omega_{\text{p}}^{\text{T}}$ , this implies that the mode *j* that contributes the majority of the oscillator strength to the $\hbar \omega_{\text{p}}^{\text{T}}$ feature likewise changes from *j* → *j* + 1 → *j* + 2 … . To illustrate this effect, the oscillator strengths of the first seven *j* modes are plotted in Figure 4 as a function of *h*.

Over the shaded region of the plot where *h* is small, the step acts as a perturbation to the dispersion of modes observed in a square well, and only the *j* = 1 mode carries any significant oscillator strength. As *h* enters the unshaded region, this is quickly no longer the case, and as shown in Figure 2(B), it becomes possible to define distinct $\omega_{\text{p}}^{\text{T}}$ and $\omega_{\text{p}}^{\text{Q}}$ modes. In this region, the oscillator strength of the *j* = 1 mode can be associated with the $\omega_{\text{p}}^{\text{Q}}$ resonance. Its continual decrease with *h* is explained considering that $N_{\text{v}}^{\text{Q}}$ also continuously decreases with *h*, and the oscillator strength of the plasmon mode is proportional to the density of charge which participates in the excitation.

The dashed line in Figure 4 is calculated as the sum of the oscillator strength of modes *j* > 1. Beyond the shaded region, the sum remains constant and closely approximates the oscillator strength of the $\omega_{\text{p}}^{\text{T}}$ feature, since for any value of *h* in this region, the only modes *j* > 1 that possess significant oscillator strength are found to lie within its linewidth, as observed for the *j* = 3 and *j* = 4 modes in Figure 3(B). With increasing step height, the mode that most contributes to the sum (or equivalently to the oscillator strength of the $\omega_{\text{p}}^{\text{T}}$ feature) is of successively increasing *j*, as previously stated. The sum remains constant because $N_{\text{v}}^{\text{T}}$ is independent of the step height.

We have demonstrated in Figure 4 that we can selectively distribute oscillator strength to different spatial modes of the electron gas in an engineered manner through control of the confining potential. As a direct consequence of the potential engineering, *j* = 4 and *j* = 6 modes, completely dark for the commonly studied case of the electron gas in a square potential, and odd *j* > 1 modes, usually possessing an oscillator strength of *j* ^−2^ times that of the *j* = 1 mode, can be made the brightest modes of the system. We highlight that these modes provide the oscillator strength for the resonance experimentally observed at 169 meV in the spectra of Figure 2(A).

## 4 Conclusions and perspectives

In this work, we have demonstrated that the electronic potential can be used as a degree of freedom to manipulate the energy and spatial mode of the excitations of a quantum confined electron gas. Simple step potential structures were studied, in which the mirror symmetry of the electronic potential was broken. The modes arising in the step potential were found to be well-described with a hybridization scheme. Importantly, the hybridization scheme reveals how to couple spatially separated plasmons with different wavevectors and how to engineer the polarization in space, thus providing a framework to guide the engineering of collective modes.

The most straightforward extension of this work is to study plasmons in other confining potentials, including those in which an additional dimension is confined, for instance, by patterning of the semiconductor growth. The dynamical modulation of the potential with the application of a strong electric field could also be explored. Our system might prove particularly interesting for engineering *χ* ^2^ nonlinearities [27], [28], [29], as well as *χ* ^3^ nonlinearities associated with epsilon-near-zero (ENZ) materials [30], [31], [32]. The latter always occur at the plasma frequency, and here we have demonstrated that we can engineer an additional one into the system. Finally, our work introduces another degree of freedom to exploit in studying polaritons or the interaction between collective excitations and a DC current [33], [34], [35], [36].

## Supplementary Material

Supplementary Material Details
