# Supplementary material for: Engineering the plasmon modes of a confined electron gas
Source: Nanophotonics. 2024 Feb 15;13(10):1851–7. doi: 10.1515/nanoph-2023-0795 (PMC11502090; doi:10.1515/nanoph-2023-0795)
Supplement: Supplementary file 1 — Supplementary Material Details [file j_nanoph-2023-0795_suppl_001.pdf]

Supplementary Material

Andrew Haky, Angela Vasanelli, Konstantinos Pantzas, Yanko Todorov, Grégoire Beaudoin, Gilles Patriarche, Isabelle Sagnes, and Carlo Sirtori*

Engineering the Plasmon Modes of a Confined Electron Gas

1 Experimental Details

All of the samples studied were grown via metalorganic chemical vapor deposition (MOCVD) on 350 µm thick semi-insulating Fe-doped InP substrates. A 5 nm Ti adhesion layer and a 150 nm gold layer were deposited directly on the epitaxial growth to serve as a mirror. The back face of the substrate was then mechanicanically polished.

We performed reflectivity experiments on the samples, as sketched in Fig. 1. Black body radiation from a Globar passes through a Fourier Transform Infrared spectrometer (FTIR) and a polarizer before it is focused onto the polished substrate side of the sample at a 72° angle of incidence. The specular reflection is collected and detected by a liquid nitrogen-cooled mercury cadmium telluride (MCT) detector. Because the plasmons are only excited by TM-polarized light, the TM polarized reflectivity spectra R^{TM} are normalized by the TE polarized reflectivity spectra R^{TE}. Polarized reference spectra, measured for light incident on a bare gold (Au) mirror, are used to normalize any polarization-dependency of the transmission of the optical set-up. The absorptivity is then found as:

$$\text{absorptivity} = 1 - \frac{R^{\mathrm{TM}}}{R^{\mathrm{TE}}} \bigg/ \frac{R^{\mathrm{Au,TM}}}{R^{\mathrm{Au,TE}}} \tag{1}$$

The 72° angle of incidence corresponds to the Brewster's angle for the InP substrate (n=3.1) and an 18° angle for the refracted light traveling inside the substrate. The plasmon spontaneous emission rate is proportional to $\sin^2\theta/\cos\theta$, where θ is the light propagation angle inside the semiconductor [1]. The 18° internal angle was chosen as a compromise between maximizing the observed contrast of the plasmon modes and avoiding additional effects which become important at larger angles, namely the blueshifting of the plasmon modes [2], and the significant radiative broadening of their linewidth

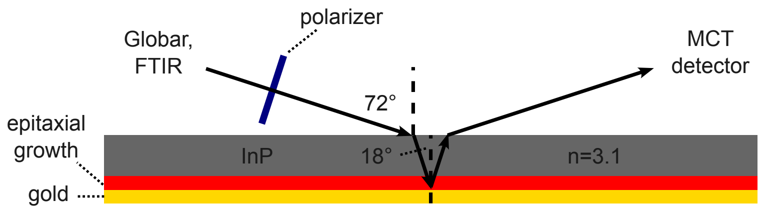

Fig. 1: The experiment is sketched.

***Corresponding author: Carlo Sirtori,** Laboratoire de Physique de l'Ecole Normale Supérieure, ENS, Université PSL, CNRS, Sorbonne Université, Université Paris Cité, 75005 Paris, France, carlo.sirtori@ens.fr; 0000-0003-1817-4554
Andrew Haky, Angela Vasanelli, Yanko Todorov, Laboratoire de Physique de l'Ecole Normale Supérieure, ENS, Université PSL, CNRS, Sorbonne Université, Université Paris Cité, 75005 Paris, France
Konstantinos Pantzas, Grégoire Beaudoin, Gilles Patriarche, Isabelle Sagnes, Center for Nanoscience and Nanotechnology, C2N UMR 9001, CNRS, Université Paris Sud, Université Paris Saclay, Palaiseau, France

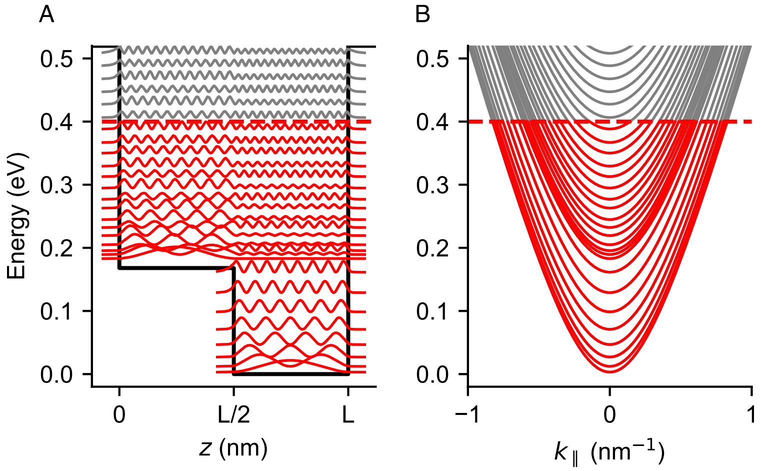

Fig. 2: (A) The square moduli of $\psi_i(z)$ are plotted, offset by E_i, for a $100\,\mathrm{nm}$ step potential with step height $h = 0.35$. (B) The dispersion of the subbands as a function of the in-plane wavevector is plotted. In both panels, the Fermi level is indicated with a dashed red line, and the states which would be occupied at zero temperature are plotted in red.

[3]. To reach larger internal angles of incidence would also require the polishing of facets onto the substrate.

2 Calculation of Plasmon Modes

The plasmon modes and their optical properties are calculated from a quantum model in the electric dipole gauge (multipolar gauge in the dipole approximation, neglecting magnetic interactions). The details of the model have been published elsewhere [4, 5]; this section is intended to recall the essential features of the model. The model is general insofar as it can be used to calculate the collective modes of the electron gas confined in an arbitrary static potential.

We begin by introducing the stationary single particle states for an electron gas in which the electronic motion is confined in the $\hat{z}$ direction. As a consequence of the confinement, the electronic states have discrete energies E_i. The electronic states are calculated within the envelope function approximation [6]. We write the z component of the envelope function associated with state E_i as $\psi_i(z)$, and for simplicity, refer to it as the electronic wavefunction. As an example, in Fig. 2(A), the square moduli of $\psi_i(z)$, calculated for a step potential, are plotted at the corresponding energies E_i. In the plane perpendicular to the confinement direction, the electrons move as free particles with in-plane momentum $\hbar k_\parallel$. Their total energy is therefore:

$$E_{i,k_\parallel} = E_i + \frac{\hbar^2 k_\parallel^2}{2m^*(E)} \tag{2}$$

The electronic states calculated for the step potential are shown in Fig. 2(B). We refer to the set of states with the same i as *subbands*. In our model, the electronic states are calculated by using a three-band $\mathbf{k}\cdot\mathbf{p}$ model, resulting in an energy-dependent effective mass $m^*(E)$ (nonparabolicity) [7].

In the next part, the collective excitations are calculated starting from the basis of stationary single particle electronic states, which immediately implies that quantum confinement is considered. We begin by introducing the bosonic raising (lowering) operators $b^\dagger_{\alpha\mathbf{q}}$ ($b_{\alpha\mathbf{q}}$) to describe the intersubband transitions $\alpha := i \to i+j$ which couple with light [8]. The photon wavevector $\mathbf{q}$ is small with respect to the typical electron wavevector, and can thus be neglected in the long wavelength approximation, so that the intersubband transition frequency is $\omega_\alpha = \omega_{i+j} - \omega_i$. This leads to an effective bosonic

Hamiltonian for the electronic excitations:

$$\hat{H}_{\mathrm{e}} = \sum_{\alpha,\mathbf{q}} \hbar\omega_\alpha b^\dagger_{\alpha\mathbf{q}} b_{\alpha\mathbf{q}} \tag{3}$$

Note that this Hamiltonian is only valid in the weak excitation regime.

The Hamiltonian of Eq. 3 describes the bare electronic excitations of the system without considering their mutual interaction through the Coulomb force (dipole-dipole interaction). It is these interactions which are responsible for the collective motion of the electron gas. To find the plasmon modes of the system, we will diagonalize the Hamiltonian which results from the addition of a term describing these interactions.

Such a term is found naturally in the interaction Hamiltonian in the electric dipole gauge, which is written:

$$\hat{H}_{\mathrm{int}} = \int \frac{1}{\epsilon_0 \varepsilon(z)} \left(-\hat{\mathbf{D}}(\mathbf{r}) \cdot \hat{\mathbf{P}}(\mathbf{r}) + \frac{1}{2}\hat{\mathbf{P}}^2(\mathbf{r}) \right) \mathrm{d}\mathbf{r} \tag{4}$$

where $\hat{\mathbf{D}}$ is the displacement field operator, $\hat{\mathbf{P}}$ is the polarization operator, and $\varepsilon(z)$ is the dielectric function. The first term of the Hamiltonian in Eq. 4 describes the light-matter interaction. In the electric dipole gauge, the displacement field is entirely transverse. It can thus be considered a free photon field totally independent from the polarization of the electron gas. The second term, quadratic in the polarization operator, describes the self-interaction of the matter polarization. It is through consideration of this term that we find the collective modes of the confined electron gas.

In terms of the operators $b^\dagger_{\alpha\mathbf{q}}$ and $b_{\alpha\mathbf{q}}$, the polarization operator can be written [4]:

$$\begin{aligned} \hat{P}_z(\mathbf{r}) &= \frac{\hbar e}{2Sm^*} \sum_{\alpha,\mathbf{q}} \frac{\xi_\alpha(z)\sqrt{\Delta N_\alpha}}{\omega_\alpha} \mathrm{e}^{\mathrm{i}\mathbf{q}\cdot\mathbf{r}_\parallel} \left(b^\dagger_{\alpha-\mathbf{q}} + b_{\alpha\mathbf{q}} \right) \\ \xi_\alpha(z) &= \psi_{i+j}(z)\frac{\partial \psi_i(z)}{\partial z} - \psi_i(z)\frac{\partial \psi_{i+j}(z)}{\partial z} \end{aligned} \tag{5}$$

where ΔN_α is the population difference between the subbands involved in a transition ΔN_α (see Sect. 3 of the Supplementary Material for the calculation of the subband populations). Importantly, the polarization operator is a function of the spatial coordinate z, as captured through the definition of $\xi_\alpha(z)$, which involves only the single particle electronic states $\psi_i(z)$. Consequently, our model leads to a nonlocal, or wavevector-dependent, description of the plasmon modes.

Using this expression for the polarization operator, the Hamiltonian for the plasmon modes can be written:

$$\begin{aligned} \hat{H}_{\mathrm{plas}} &= \hat{H}_{\mathrm{e}} + \int \frac{1}{2\epsilon_0\varepsilon(z)} \hat{\mathbf{P}}^2(\mathbf{r})\,\mathrm{d}\mathbf{r} \\ &= \sum_{\alpha,\mathbf{q}} \hbar\omega_\alpha b^\dagger_{\alpha\mathbf{q}} b_{\alpha\mathbf{q}} + \frac{e^2}{2\epsilon_0\varepsilon} \sum_{\alpha,\beta,\mathbf{q}} S_{\alpha\beta} \sqrt{\Delta N_\alpha \Delta N_\beta} \left(b^\dagger_{\alpha\mathbf{q}} + b_{\alpha-\mathbf{q}} \right) \left(b^\dagger_{\beta-\mathbf{q}} + b_{\beta\mathbf{q}} \right) \end{aligned} \tag{6}$$

where

$$S_{\alpha\beta} = \frac{1}{\hbar\omega_\alpha} \frac{1}{\hbar\omega_\beta} \left(\frac{\hbar^2}{2m^*} \right)^2 \int_{-\infty}^{\infty} \xi_\alpha(z)\xi_\beta(z)\,\mathrm{d}z \tag{7}$$

The coupling coefficients $S_{\alpha\beta}$ describe the spatially dependent coupling between any two transitions α and β.

The quadratic Hamiltonian is diagonalized in two steps [5]. In the first step, the Hamiltonian which describes the coupling only between identical transitions $\alpha = \beta$ is diagonalized via a Bogoliubov transformation. Bosonic operators, written as linear combinations of $b^\dagger_{\alpha\mathbf{q}}$ and $b_{\alpha\mathbf{q}}$, are introduced to describe the normal modes of this Hamiltonian, which are the *intersubband plasmons*. In the second step, the full Hamiltonian of Eq. 6 is rewritten in terms of these operators and is diagonalized via

a second Bogoliubov transformation, such that the full Hamiltonian of Eq. 6 is written in terms of bosonic operators $P^\dagger_{n\mathbf{q}}$, $P_{n\mathbf{q}}$ for the *multisubband plasmons* n with excitation frequency Ω_n:

$$\hat{H}_{\text{plas}} = \sum_{n,\mathbf{q}} \hbar\Omega_n P^\dagger_{n\mathbf{q}} P_{n\mathbf{q}} \tag{8}$$

The bosonic operators for the multisubband plasmons can be expressed as linear combinations of the original operators $b^\dagger_{\alpha\mathbf{q}}$ and $b_{\alpha\mathbf{q}}$.

The polarization operator can now be expressed in terms of the multisubband plasmon operators:

$$\hat{P}_z(\mathbf{r}) = \sum_{n,\mathbf{q}} \frac{J_n(z)}{\Omega_n} \mathrm{e}^{\mathrm{i}\mathbf{q}\cdot\mathbf{r}_\parallel} \left(P_n + P^\dagger_n\right) \tag{9}$$

$$J_n(z) = \frac{e\hbar}{2\sqrt{S}m^*}\Omega_n \sum_\alpha \frac{\xi_\alpha(z)\sqrt{\Delta N_\alpha}}{\sqrt{\omega_\alpha \tilde{\omega}_\alpha}} X_{\alpha_n} \tag{10}$$

where S is the area of the system, X_{α_n} is an $N \times N$ matrix determined by the N eigenvectors found upon diagonalizing Eq. 6, and $\tilde{\omega}_\alpha = \sqrt{\omega_\alpha^2 + \omega_{\mathrm{p}_\alpha}^2}$, where $\omega_{\mathrm{p}_\alpha}^2 = \frac{2e^2\Delta N_\alpha}{\hbar\epsilon_0\varepsilon} S_{\alpha\alpha}$ is a plasma frequency for the electronic density ΔN_α which partakes in the transition α. The z-dependence of the polarization operator is entirely captured in the definition of the current density $J_n(z)$. It is this quantity which is referred to as a microcurrent in the main text.

The absorption coefficient, calculated from Fermi's golden rule in the perturbative light-matter coupling regime, is obtained by integrating the contributions of the different multisubband plasmon current densities, and can be written as [5]:

$$\alpha_{2\mathrm{D}} = \frac{S}{\epsilon_0 c\sqrt{\varepsilon}} \sum_n \frac{1}{\Omega_n} \left| \int_{-\infty}^{\infty} J_n(z)\,\mathrm{d}z \right|^2 \mathcal{L}\left(\omega - \Omega_n\right) \tag{11}$$

where $\mathcal{L}\left(\omega - \Omega_n\right)$ is a Lorentzian centered at the plasmon frequency. From this expression, it is apparent that the quantity $\frac{1}{\Omega_n}\left|\int_{-\infty}^{\infty} J_n(z)\,\mathrm{d}z\right|^2$ can be considered an effective oscillator strength for the plasmon mode n. The absorptivity spectra reported in the main text are calculated nonperturbatively (without making the rotating wave approximation) as described in [9], by solving the quantum Langevin equations in the input-output formalism.

In the description presented here, the collective modes of the system arise due to the pairwise Coulomb interaction between all optically allowed single particle transitions. In the particular case of a square well potential, the coupling coefficent $S_{\alpha\beta} = S_{i\to i+j, i'\to i'+j'}$ is only nonzero for $j = j'$, so that only transitions which occur between the same number of levels j couple. Then, the Hamiltonian can be independently diagonalized for each subspace j, yielding j plasmon modes [10]. As described in the main text, these modes correspond to standing polarization waves in the z direction: $J_j(z) \propto \sin\frac{j\pi z}{L}$ where L is the length of the well.

3 Effective Plasma Frequencies

In this section, we describe in greater detail the calculation of the effective plasma frequencies $\omega_\mathrm{p}^\mathrm{T}$ and $\omega_\mathrm{p}^\mathrm{Q}$. They are defined as:

$$\omega_\mathrm{p}^\mathrm{T} = \sqrt{\frac{e^2 N_\mathrm{v}^\mathrm{T}}{m^*\varepsilon_\infty\epsilon_0}} \quad \text{and} \quad \omega_\mathrm{p}^\mathrm{Q} = \sqrt{\frac{e^2 N_\mathrm{v}^\mathrm{Q}}{m^*\varepsilon_\infty\epsilon_0}} \tag{12}$$

where e is the elementary charge, N_v^T (N_v^Q) is the volume electronic density calculated over the ternary (quaternary) region of the step structure (described in greater detail below), m^* is the free electron effective mass, $\epsilon_\infty = 3.1$ is the high frequency dielectric constant, and ϵ_0 is the vacuum permittivity.

The effective mass $m^* = 0.095m_0$, where m_0 is the electron mass, was determined by fitting the curves of ω_p^T and ω_p^Q to the experimental data plotted in Fig. 2(B) in the main text. This value is reasonable: the effective masses of $In_{0.53}Ga_{0.47}As$ and $Al_{0.44}In_{0.52}As$ at their conduction band minima are $m^*_{InGaAs} = 0.043m_0$ and $m^*_{AlInAs} = 0.072m_0$. The electrons which participate in the collective excitations have energies close to the Fermi level (a consequence of Pauli blocking), which in our experiment lies approximately 0.4 eV above the InGaAs conduction band minimum. At this energy, the electron effective mass is considerably larger due to the strong nonparabolicity of the electronic band structure.

We now turn to the calculation of the volume electronic densities N_v^T and N_v^Q. For an energy-dependent effective mass, the 2D density of states of subband i takes the form:

$$\rho_i^{2D}(E) = \frac{1}{\pi\hbar^2}\left(m^* + E\frac{dm^*}{dE}\right) \tag{13}$$

The sheet density n_i (units of $[\text{length}]^{-2}$) of carriers within a subband i is given by the integral of the Fermi-Dirac distribution function, $f_{FD}(E)$, times the 2D density of states of the subband [11]:

$$n_i = \int_{E_i}^{\infty} f_{FD}(E)\rho_i^{2D}(E)\,dE \tag{14}$$

The sheet density δn of an infinitesimal region of thickness δz within the confining potential can be found as:

$$\delta n = \sum_i n_i \left|\psi_i(z)\right|^2 \delta z \tag{15}$$

The volume electronic densities N_v^T and N_v^Q are found by dividing the sheet densities calculated over the respective quaternary $[0, L/2]$ and ternary $[L/2, L]$ intervals by the interval length $L/2$:

$$N_v^Q = \frac{1}{L/2}\int_0^{\frac{L}{2}}\left(\sum_i n_i\left|\psi_i(z)\right|^2\right)dz \quad \text{and} \quad N_v^T = \frac{1}{L/2}\int_{\frac{L}{2}}^{L}\left(\sum_i n_i\left|\psi_i(z)\right|^2\right)dz \tag{16}$$

The occupancy of the subbands, and thus the electronic density calculated over any interval, is determined by the temperature T and the Fermi energy E_F which enter the Fermi-Dirac distribution in Eq. 14. Since the experiments described in the text were performed at room temperature, we calculate the subband populations for T=300 K.

We find that the experimental results are best modeled (both for the effective plasma frequency approach, and the spectra calculated using the full quantum model plotted with the colormap in Fig. 2(B) of the main text) by fixing the Fermi level at 0.4 eV above the InGaAs conduction band minimum as the step height is varied. With increasing step height, the density of states below the fixed Fermi level decreases, and consequently, the volume electronic density evaluated over the entire structure decreases from $N_v = 1.73 \times 10^{19}\,\text{cm}^{-3}$ for $z = 0.13$ to $N_v = 1.43 \times 10^{19}\,\text{cm}^{-3}$ for $z = 0.35$. While all of the structures were grown with the same nominal dopant density, it is expected that the actual electronic density should decrease with increasing step height, as the incorporation of dopants in the quaternary material is less efficient with increasing Al content.

References

[1] T. Laurent, Y. Todorov, A. Vasanelli, A. Delteil, C. Sirtori, I. Sagnes, and G. Beaudoin, "Superradiant Emission from a Collective Excitation in a Semiconductor," *Physical Review Letters*, vol. 115, no. 18, p. 187402, Oct. 2015.

[2] G. Frucci, S. Huppert, A. Vasanelli, B. Dailly, Y. Todorov, G. Beaudoin, I. Sagnes, and C. Sirtori, "Cooperative Lamb shift and superradiance in an optoelectronic device," *New Journal of Physics*, vol. 19, no. 4, p. 043006, Apr. 2017.

[3] S. Huppert, A. Vasanelli, T. Laurent, Y. Todorov, G. Pegolotti, G. Beaudoin, I. Sagnes, and C. Sirtori, “Radiatively Broadened Incandescent Sources,” *ACS Photonics*, vol. 2, no. 12, pp. 1663–1668, Dec. 2015.

[4] Y. Todorov and C. Sirtori, “Intersubband polaritons in the electrical dipole gauge,” *Physical Review B*, vol. 85, no. 4, p. 045304, Jan. 2012.

[5] G. Pegolotti, A. Vasanelli, Y. Todorov, and C. Sirtori, “Quantum model of coupled intersubband plasmons,” *Physical Review B*, vol. 90, no. 3, p. 035305, Jul. 2014.

[6] G. Bastard, *Wave Mechanics Applied to Semiconductor Heterostructures*, ser. Monographies de Physique. Les Ulis Cedex, France : New York, N.Y: Les Editions de Physique ; Halsted Press, 1988.

[7] C. Sirtori, F. Capasso, J. Faist, and S. Scandolo, “Nonparabolicity and a sum rule associated with bound-to-bound and bound-to-continuum intersubband transitions in quantum wells,” *Physical Review B*, vol. 50, no. 12, pp. 8663–8674, Sep. 1994.

[8] C. Ciuti, G. Bastard, and I. Carusotto, “Quantum vacuum properties of the intersubband cavity polariton field,” *Physical Review B*, vol. 72, no. 11, p. 115303, Sep. 2005.

[9] S. Huppert, A. Vasanelli, G. Pegolotti, Y. Todorov, and C. Sirtori, “Strong and ultrastrong coupling with free-space radiation,” *Physical Review B*, vol. 94, no. 15, p. 155418, Oct. 2016.

[10] A. Vasanelli, S. Huppert, A. Haky, T. Laurent, Y. Todorov, and C. Sirtori, “Semiconductor Quantum Plasmonics,” *Physical Review Letters*, vol. 125, no. 18, p. 187401, Oct. 2020.

[11] P. Harrison, *Quantum Wells, Wires and Dots: Theoretical and Computational Physics*, 3rd ed. West Sussex, England ; Hoboken, NJ: Wiley, 2009.
